# Array CGH Phylogeny: How accurate are Comparative Genomic Hybridization-based trees?

**DOI:** 10.1186/1471-2164-12-487

**Published:** 2011-10-06

**Authors:** Luz B Gilbert, Takao Kasuga, N Louise Glass, John W Taylor

**Affiliations:** 1 Laboratoire de Recherche en Sciences Végétales, UMR CNRS-Université Paul Sabatier 5546, Chemin de Borde Rouge - Auzeville 31326, Castanet Tolosan, France; 2 USDA ARS, Plant Pathology Department, UC Davis, Davis, CA, 95616, USA; 3 Department of Plant and Microbial Biology, UC Berkeley, Berkeley, CA 94720, USA

## Abstract

**Background:**

Array-based Comparative Genomic Hybridization (CGH) data have been used to infer phylogenetic relationships. However, the reliability of array CGH analysis to determine evolutionary relationships has not been well established. In most CGH work, all species and strains are compared to a single reference species, whose genome was used to design the array. In the accompanying work, we critically evaluated CGH-based phylogeny using simulated competitive hybridization data. This work showed that a limited number of conditions, principally the tree topology and placement of the reference taxon in the tree, had a strong effect on the ability to recover the correct tree topology. Here, we add to our simulation study by testing the use of CGH as a phylogenetic tool with experimental CGH data from competitive hybridizations between *N. crassa *and other *Neurospora *species. In the discussion, we add to our empirical study of *Neurospora *by reanalyzing of data from a previous CGH phylogenetic analysis of the yeast *sensu stricto *complex.

**Results:**

Array ratio data for *Neurospora *and related species were normalized with loess, robust spline, and linear ratio based methods, and then used to construct Neighbor-Joining and parsimony trees. These trees were compared to published phylogenetic analyses for *Neurospora *based on multilocus sequence analysis (MLSA). For the *Neurospora *dataset, the best combination of methods resulted in recovery of the MLSA tree topology less than half the time. Our reanalysis of a yeast dataset found that trees identical to established phylogeny were recovered only by pruning taxa - including the reference taxon - from the analysis.

**Conclusion:**

Our results indicate that CGH data can be problematic for phylogenetic analysis. Success fluctuates based on the methods utilized to construct the tree and the taxa included. Selective pruning of the taxa improves the results - an impractical approach for normal phylogenetic analysis. From the more successful methods we make suggestions on the normalization and post-normalization methods that work best in estimating genetic distance between taxa.

## Background

Microarray-based Comparative Genomic Hybridization (Array CGH) for two-color array platforms uses DNA samples from a reference individual and a test individual, each labelled with a different fluorescent dye, and competitively hybridizes them to an array composed of immobilized DNA fragments based on genomic sequence of the reference individual [1-4]. Array CGH produces abundant information about genetic distance for all genes between pairs of individuals. This ability to estimate genetic distance for all genes in one assay has made array CGH an attractive tool for phylogenetic analysis. Several studies have used array CGH to compare bacterial species [5-10], and in one case both human and bovine data [11], to deduce evolutionary relationships from cluster analysis. Other studies, also mostly in bacterial systems, applied distance and parsimony tree-building techniques to construct phylogenies from microarray data [12-20].

A complication that has not been addressed in these studies involves the use of one species to design the array, which requires that all competitive hybridizations have as one partner the same reference species. This situation has been termed "unbalanced gene content" [21] or, as we will refer to it, the single reference design. Use of a single reference individual is at odds with traditional sequence-based phylogenetic analysis, where all pairwise comparisons among the taxa are used for tree construction. An underlying assumption of array CGH phylogeny is that the massively parallel nature of microarrays, where genes number in the thousands, will provide enough phylogenetic signal to resolve a tree, even when the microarray is based on a single reference taxon.

This approach has been applied to bacterial species with largely clonal reproduction, for example studies by Dagerhamn, Wan, and Guidot [18,20,22] reported uses of CGH for a subset of genes to recover clusters of bacteria concordant with previously published MLSA phylogenies. The work of Solheim *et al *directly compared MLST with aCGH trees for *Enteroccocus *species with the goal of defining lineage-specific genes [23]. Although the biology of bacteria differs from that of fungi like *Neurospora *or yeast in that bacterial horizontal gene transfer is not the same as eukaryotic mating and meiosis, we wanted to determine if CGH would be as useful with eukaryotic microbes as it appeared to be with bacteria.

In the accompanying study we examined this question using an *in silico *approach. While it was sometimes possible to recover the topology used to initiate the simulation, it was possible for only a very restricted set of conditions involving the underlying tree topology. Key parameters affecting success were the position in the topology of the reference taxon, and the method of phylogenetic analysis.

To challenge our *in silico *findings with experimental data, we developed experimentally derived CGH data for *Neurospora*, and used it to test CGH as a tool for phylogenetic analysis that could be applied to any group of genetically isolated taxa with any combination of clonal and recombining reproduction. Using the 70 mer expression array designed for *Neurospora crassa *[24,25], we compared seven species of *Neurospora*, as well as the related species *Podospora anserina *and *Sordaria macrospora*. The relationships of these nine species have been established by phylogenetic analyses of multiple DNA sequences (multilocus sequence analysis, MLSA), which has provided a well supported tree spanning closely related *Neurospora *species and representatives of their neighboring genera [26-28]. The close relatedness of these taxa — and the fact that they are not subject to large amounts of fluctuation in gene content — makes them excellent candidates for successful aCGH phylogeny.

Based on published accounts, we used eight general methods of phylogenetic analysis of aCGH data. These approaches, which are detailed in the methods section, encompass different methods of data normalization and post-normalization, two basic tree construction methods (Neighbor-Joining and Parsimony), and three more sophisticated methods of processing CGH ratio data (BAGEL, MPP, and GACK - see Additional File 1: table S1). To compare the phylogenies inferred from array CGH analysis to those based on MLSA, we used the symmetric (SymD) and agreement subtree (D1) tree-to-tree distance metrics [26-28] implemented in the program PAUP [29].

We show that CGH cannot be counted on to recover the established multilocus phylogeny for *Neurospora *[26]. We also find, in our reanalysis of yeast CGH data [17], that CGH cannot be counted on to recover the MLSA phylogeny for *Saccharomyces sensu stricto *species. A good phylogenetic method should be able to recover phylogenies in cases where there is sufficient phylogenetic signal in the genetic or genomic variation to produce a well-supported phylogeny. Our application of phylogenetic methods to these two sets of empirical CGH data does nothing to contradict the conclusions of our accompanying analysis of simulated data [Gilbert et al.: Array Comparative Genomic  Hybridizations: Assessing the ability to recapture evolutionary  relationships using an in silico approach. BMC Genomics 2011 12:456]. In fact, our results suggest that the noise inherent in array CGH data further compromises use of these data for phylogenetics.

## Methods

### DNA Preparation and Hybridization

Fungal strains used in this study are listed in Table 1. *Neurospora *strains were obtained from the Fungal Genetics Stock Center [30], *Sordaria macrospora *was generously provided by the Kück lab (Lehrstuhl fur Allgemeine Botanik, Ruhr-Universitat, Bochum, Germany), while *Podospora anserina *was provided by Philippe Silar of the Institut de Génétique et Microbiologie (Université de Paris-Sud XI/CNRS).

**Table 1:** Strains used in this study

**Identifier**	**Other ID**	**Species**	**Origin**	**Mating Type**	**Comment**
FGSC 2489		*Neurospora crassa *A	Louisiana	A	conidiating taxa
FGSC 8781	D21	*Neurospora intermedia*	Florida	A	conidiating taxa
FGSC 8858	D98	*Neurospora crassa *C	Tamil Nadu, India	A	conidiating taxa
FGSC 8813	D53	*Neurospora sitophila*	Thailand	A	conidiating taxa
FGSC 8775	D15	*Neurospora tetrasperma*	Hawaii	a	conidiating taxa
FGSC 8906	D146	*Neurospora discreta*	New Mexico	a	conidiating taxa
FGSC 1889		*Neurospora terricola*		homothallic	homothallic non conidiating
S48977		*Sordaria macrospora*		homothallic	K strain, courtesy of the Kuck lab, Ruhr-Universität Bochum
S strain		*Podospora anserina*		+	courtesy of Phiippe Silar of the Institut de Génétique et Microbiologie Université de Paris-Sud

Genomic DNA was isolated with the DNeasy Plant Tissue kit (Qiagen, Valencia, CA) with the following modifications: tissue was lyophilized, ground, and then incubated at 65 C for an hour with 50 mM Tris-HCl, 50 mM EDTA, 3% SDS solution with 100 μl of Proteinaise K (20 mg/ml). This was followed by chloroform:isoamyl alcohol extraction. The aqueous phase was added to the Qiagen extraction buffer and extraction proceeded according to the manufacturer's instructions.

Genomic DNA was sheared mechanically with a target range of 1 kb using a Hydroshear^® ^(GeneMachines™, San Carlos, CA). Test species and reference species were labelled using the BioPrime^® ^Plus Array CGH Indirect Genomic Labeling kit (Invitrogen, Carlsbad, CA). A full-genome 70 mer oligonucleotide microarray representing 10,918 individual elements was constructed as described for a partial [24] and the full genome array [25]. Slides were treated to minimize background fluorescence with the Pronto!™ Background Reduction Kit according to the manufacturer's instructions (Corning, Lowell, MA), then pre-hybridized and hybridized as described [31] with several modifications, which are published online at http://www.yale.edu/townsend/Links/ffdatabase/downloads.html.

Images were scanned with an Axon GenePix 4000 B scanner (Molecular Devices Corporation, Sunnyvale, CA). GenePix Pro 6 software was used to quantify hybridization signals. Bad spots were flagged automatically by GenePix software and each slide was manually inspected. Each species comparison was done in at least quadruplicate with dye swaps.

### Data Filtration and Normalization

Data were normalized for non-biological variation related to printing and hybridization of spotted microarrays by four methods. The first normalization, linear and based on the ratio of means (Acuity 4.0, Molecular Devices Corporation, Sunnyvale, CA), used a set of twelve control spots with no known sequence polymorphisms between *N. crassa *and *N. discreta *to standardize the ratio data (unpublished data). For the second normalization, print-tip lowess (locally weighted linear regression, Acuity 4.0), an additional percent pixel saturation cutoff and a roundness score were used to filter the initial dataset according to the Acuity manufacturer's recommendations. The third normalization method is a different implementation of loess (locally weighted quadratic regression) and the fourth is robust spline (regression spline with empirical Bayes shrinkage), both from the R package Limma [32-34].

In addition to the four kinds of normalization, filtered according to the criteria discussed in the methods, an additional filtering criterion was applied for a duplicate subset of the linear and lowess normalizations, where a spot had to scored as present in at least 40% of the slides to be included. These additionally filtered data sets are referred to as the "40% present" set while the rest are referred to as the standard set. All were imported to R to calculate correlation and Euclidean distance matrices. PAUP Neighbor-Joining trees were constructed from these matrices.

### Distance and Binary Matrix Calculation

Using the functions cor() and dist() from the R stats package [35], the Pearson correlation or Euclidean distance was calculated from mean or median ratio values to make a distance matrix. Note that this step does not require discretizing the ratio values prior to the distance calculation. Euclidean and correlation-based distance matrices (1-correlation) were used for Neighbor-Joining tree construction. Distance matrices were calculated for normalized filtered ratio values and for BAGEL (Bayesian Analysis of Gene Expression Level) estimates of hybridization level.

To convert continuous distances to binary characters (1,0), we used the program GACK, Genome Composition analysis by Charles Kim, to implement a "genomotyping" method. This program employs a dynamic cutoff based on the signal ratio distribution to classify genes as present or absent [36]. The exact value of the cutoff could be modified by changing the value of the expected probability of being present (EPP) within the range of 0 to 100% [36]. For example, a 50% EPP value, calculated from the distribution of the ratio data, categorizes all genes with a 50% or greater chance of being present as conserved (1), and those with a less than 50% chance as diverged (0). These binary matrices were used for parsimony tree construction.

### Phylogenetic Analysis

Phylogenies were created from distance data by the Neighbor-Joining (NJ) algorithm from PAUP version 4.0b10 [29]. Phylogenies were created from discrete data with the parsimony analysis using a heuristic search with 500 replicates, followed by construction of a 50% majority rule consensus tree (PMR) also in PAUP [29].

### BAGEL

We used Bayesian analysis of gene expression levels (BAGEL) because it estimates hybridization levels for the reference species differently than all of the other methods that we employed, except MPP. Typically, hybridization values for the reference species are taken from hybridizations between the reference species and itself. However, with BAGEL, for each gene, the ratio data from all the hybridizations involving the reference and test species are used to estimate a relative hybridization level for each species, including an extrapolated value for the reference species [37,38]. For distance phylogenetic analyses, the BAGEL output was converted to Euclidean and correlation-based distance matrices as before. For parsimony phylogenetic analyses, the ratio data were converted to binary characters by binning the ratio values in the taxon-by-gene matrix in one of the four quartiles (Q1, Q2, Q3 or Q4) and choosing to score presence (1) for genes with values in Q4, Q4-Q3, or Q4-Q2 and absence (0) for genes with values in lower quartiles (Q3-Q1, Q2-Q1 or Q1, respectively which correspond to the negative or smaller hybridization ratios). This binary data set made from BAGEL-treated data could then be compared to the one made by applying GACK to the filtered, normalized data.

### MPP

In addition to genomotyping with GACK, we utilized a second genomotyping program -the Microarray to Phylogeny Pipeline (MPP) [21]. MPP is an all-inclusive pipeline that uses CGH hybridization data, converts it to binary and uses those values to create a distance matrix for tree construction.

GPR files from the GenePix program were input into MPP and replicate spots were averaged according to species. The ratio data were filtered and then log_-_transformed or transformed with the inverse hyperbolic sine (arsinh) function [35,39]. Following transformation, the data were binned with the EPP method or the Bayesian Probability of Presence (BPP), to determine thresholds for the presence or absence of a probe. A binwidth setting of .05 and the experimental binwidth option (designated as norm and exp) were applied to the data during the binning process. Binned matrices were exported from the program for parsimony phylogenetic analysis by PAUP (not a recommendation of the program's creators) or were used to calculate a pairwise distance matrix using the CGHdist method implemented in MPP. This method employs a death process to model loss of genes in a set of related taxa and is meant to compensate for the "unbalanced gene content" that results from having a single reference taxon for array CGH [21]. During this process MPP extrapolates a value for the reference from the comparisons. This pairwise distance matrix was then used for Neighbor-Joining tree construction.

### Tree to tree distance metric quantification

To quantitatively assess the differences between CGH-derived and MLSA trees, we compared the MLSA topology for the nine Sordariomycete fungi [26-28] to the CGH derived trees using two tree-to-tree distance metrics, symmetric distance (SymD) and agreement subtree (D1), implemented in PAUP [29]. The symmetric distance, SymD, determines the number of branches that must be rearranged or collapsed to make two topologies identical. If the topologies are identical the step size is 0. One step indicates a single collapsed branch and a rearrangement between two taxa is scored as two steps [40]. The agreement subtree metric, D1, counts the number of taxa that must be pruned to make two topologies identical [41]. The symmetric distance is reported by default while values for D1 are available in the Additional Files 2, 3, and 4: table S2, S3, and S4.

## Results

Almost all of the steps needed to process array CGH ratio data for phylogenetic analysis can influence the result. These include the filtering, normalization and tree-building procedures applied to the data. Using empirical data, we tested the effect of different analytical approaches on distance and parsimony analysis. Different methods of normalization combined with mean or median hybridization values for each species were used. For Neighbor-Joining analysis, different metrics for converting normalized intensity ratios to genetic distances were used, as well as different thresholds for converting ratios to discrete character data for parsimony analysis. One such method was a probabilistic method for converting ratios to discrete character data (GACK) and the other was a self-contained work-flow (MPP), which converts raw CGH data to data suitable for phylogenetic analysis. An alternative to average or median CGH ratio values, BAGEL (a Bayesian approach to estimate a representative hybridization value for each species), was also tested.

**Figure 1:**
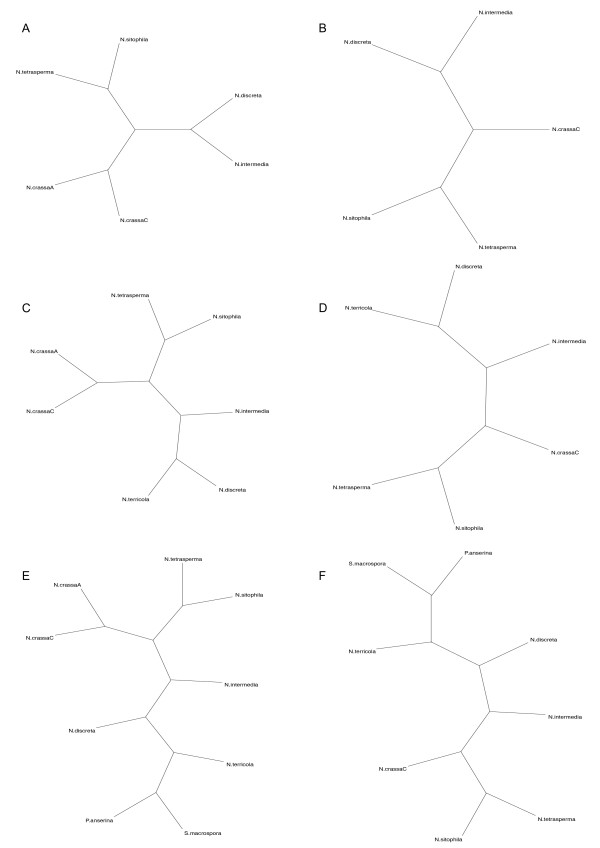
**Desired topology as cladograms**. The figure shows six different trees, different permutations of the *Neurospora *topology that we attempted to recover from CGH data using a variety of methods. These trees are broken down into three groupings of taxa, CON (Figures 1A and 1B), NEU (Figures 1C and 1D), and ALL (Figures 1E and 1F). These are shown with and without the reference *N. crassa *A as some methods performed better without the reference taxon included. For this reason both versions of each taxon grouping were used to construct CGH phylogenies. Note that these cladograms do not reflect true branch length distances.

To consider the effect of evolutionary distance among taxa on the analyses, we grouped taxa into three datasets that varied in combined genetic distance. The CON set consisted of the six most closely related outbreeding individuals of *Neurospora *(*N. crassa *A, *N. crassa *C, *N. sitophila, N. tetrasperma, N. intermedia*, and *N. discreta*). The NEU set is the CON set plus *N. terricola*, an obligately self-fertilizing relative. The ALL taxa set is the NEU set plus two more distant relatives, *Sordaria macrospora *and *Podospora anserina*. Phylogenetic trees for these taxon sets, based on multilocus sequence analyses (MLSA; [26-28]) are shown in Figure 1, with and without the reference taxon as indicated. As detailed in the methods, in all analyses, we present results with and without the reference taxon because some analyses are confounded by the zero distance between the reference taxon and itself.

Summaries of our analyses given below are supported by analyses in the additional material as follows: Analysis of filtered normalized data for NJ (Additional File 1: table S1A) and parsimony after GACK processing (Additional File 1: table S1B). Analysis of Bayesian-based (BAGEL) treatments for NJ (Additional File 1: table S1C) and parsimony (Additional File 1: table S1D). Parsimony analyses using MPP (Additional File 1: table S1F). MPP was also used to implement a likelihood approach with Neighbor-Joining to compensate for the single reference design (Additional File 1: table S1E).

### Neighbor-Joining Analysis Normalized data, (Figure 2A &2B, Additional File 2: table S2)

**Figure 2:**
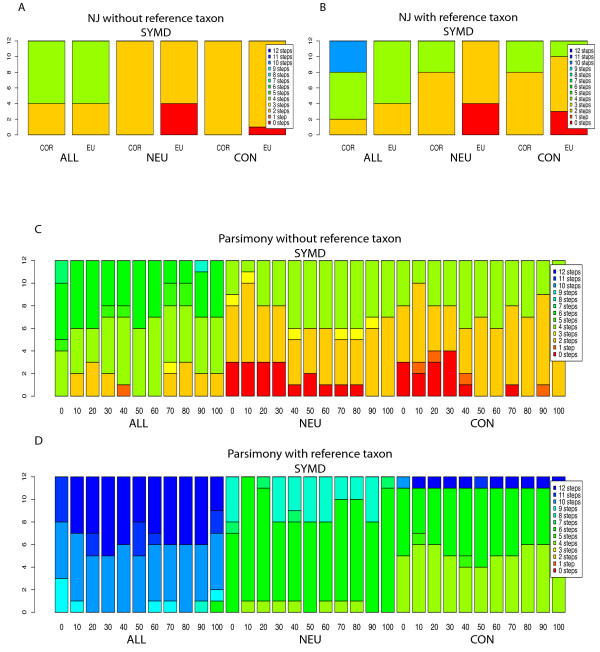
**Results for NJ and Parsimony analysis of normalized ratio data**. These stacked histograms in this figure represent the SymD measures (symmetric distance away from the desired topology) for the Neighbor-Joining (Figures 2A and 2B) and parsimony (Figures 2C and 2D) CGH trees constructed from the ACUITY and Limma-based normalizations. Each stack represents the twelve iterations of the four different normalization procedures, detailed in Additional File 1: table S1. In histograms 2A and 2B, a correlation (COR) or a Euclidean distance matrix (EU) was calculated for each of the twelve iterations described using R and input into PAUP in order to construct NJ trees for the ALL, NEU, and CON taxa sets excluding (2A) and including (2B) the reference taxa, respectively. Figures 2C and 2D show stacked histograms for the corresponding Parsimony Majority Rule consensus (PMJ) trees binned with the GACK method. For each stack, the EPP threshold was varied in 10% increments from 0 to 100%. The same data is given in table form in Additional File 2: table S2.

In no case did analysis of the ALL taxa set by Neighbor-Joining of CGH data that had been simply normalized produce the same tree as MLSA tree (Figure 2A). The most successful analyses produced trees at least two steps longer than the MLSA tree as judged by the SymD (symmetric distance) metric. These most successful analyses used NJ with either Pearson's correlation coefficient or Euclidean distance and with or without the reference taxon, and with loess (Limma), robust spline or linear normalization, but never with lowess (Acuity) normalization. Taking the mean or median of normalization values had no effect on the outcome and neither did employing the additional 40% filter.

With the NEU taxa set, the MLSA tree was recovered perfectly by NJ using Euclidean distance, but only with linear normalization based on the ratio of means. There was no effect of including or excluding the reference taxon, using the mean or median value for each gene, or adding the additional 40% filter. Clearly, exclusion of the distant taxa, *Sordaria *and *Podospora*, had a positive effect on the analyses, but only with this combination of methods.

With the CON taxa set, the MLSA tree was recovered less frequently. Here, again, the most robust result (0 steps away) was by NJ analysis using Euclidean distance of data linearly normalized by the linear ratio of means. However, results were better when the reference taxon was included and the additional 40% filter was omitted on mean values for each gene. Again, inclusion or exclusion of the more divergent taxa had the largest effect on recovery of the MLSA tree and trees with topologies close to the MLSA tree were found only with a narrow combination of methods.

### Parsimony Analysis Normalized data, (Figure 2C &2B, Additional File 2: table S2)

For the ALL dataset, with or without the reference, no trees with topologies identical to the MLSA tree were produced for any normalization. With the reference taxon included, the averaged loess and median of the spline normalization give trees two to four steps distant for most values of the %EPP cutoff. The lowess trees were 1 to 10 steps longer than the MLSA tree, depending on the values of the %EPP. Trees that included the reference taxon were substantially worse (see Additional File 2: table S2).

With the NEU taxon set, several of the thresholds based on %EPP resulted in trees with topologies identical to the MLSA tree when the reference taxon was excluded. These trees identical to MLSA trees included those made using the averaged values of the loess and the median values of the spline normalizations, and many of the percent EEP values using averaged linear normalization. Adding the additional 40% filter had a negative effect such that only the 50% EPP threshold gave the MLSA tree topology. With the reference taxon included in the analysis, the MLSA tree topology was not recovered as judged by the SymD metric (no closer than six steps) or the D1 metric (as close as one step, see Additional File 2: table S2).

For the CON dataset, excluding the reference, the averaged values of the linear and loess normalizations recovered the MLSA tree topology in four and five of the eleven percent EPP thresholds respectively. The median and average values of the robust spline normalization were also successful in capturing the MLSA tree. Again, the additional 40% filter resulted in poorer trees overall. The remaining iterations of the data were two to four steps away. Including the reference species gave trees that were no closer to the MLSA tree than four steps and then only for the spline and loess normalizations.

### NJ after Bayesian estimation of a relative hybridization level (Figure 3A and 3B, Additional File 3: table S3)

For the ALL taxa set, with and without the reference species, the MLSA tree topology was not recovered with either the Euclidean or correlation-based metric. The closest approximation of the MLSA tree was achieved by the robust spline and loess normalizations (two steps longer by both the Euclidean and correlation distance metrics). For the correlation metric, including the reference species had no effect on results. However, for the Euclidean metric, including the reference species in some cases increased the length as compared to the MLSA tree by from two to six steps.

For the NEU dataset, the Euclidean distance metric captured the MLSA tree for both the spline and loess normalizations while the correlation metric did so solely with the robust spline normalization. This result was found regardless of whether or not the reference taxon was included. For the CON dataset, the correlation metric outperformed the Euclidean metric by capturing the MLSA tree with almost all approaches (except the lowess normalization) regardless of whether the reference taxon was included or excluded. The Euclidean metric recovered the MSLA tree topology with fewer combinations of approaches, and performed worse when the reference taxon was included (Limma spline normalization moved from no to two steps distant).

One noteworthy result with BAGEL NJ trees is that, unlike the other methods tested, the results are far less sensitive to inclusion or exclusion of the reference taxon. This insensitivity is presumably due to BAGEL's extrapolation of the reference value, which appears to be a more robust way of including the reference taxon than including self-self controls for tree construction.

### Bagel Parsimony (Figure 3C and 3D, Additional File 3: table S3)

**Figure 3:**
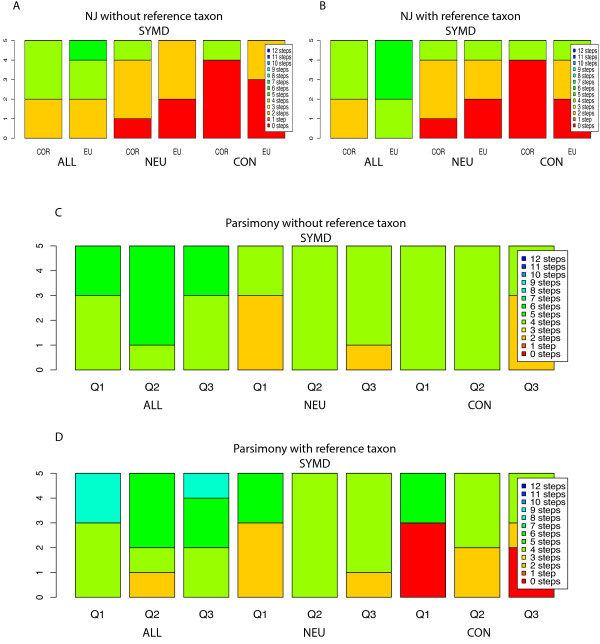
**Results for NJ and Parsimony analysis of Relative Bayesian Estimated Hybridization Levels**. Figure 3 show the stacked histograms of the SymD measures for Neighbor-Joining tree construction of the ACUITY and Limma-based normalizations processed with the BAGEL program. Five trees are represented in each stack, constructed from each of the four normalizations done and the additional linear normalization based on the ratio of the medians (see Additional File 1: table S1). Only these five datasets were input into BAGEL as it only accepts log-transformed data. Figures 3A and 3B show the results for the NJ algorithm excluding and including *N. crassa *A respectively for the symmetric distance. Figures 3C and 3D show the SymD measures from parsimony analysis of the BAGEL-estimated values converted to binary matrices as described in the methods. The same data is given in table form in Additional File 3: table S3.

For parsimony analysis, the BAGEL estimates of hybridization levels described above were binned at the first, second, and the third quartile. For the ALL dataset, no method of analysis recovered the MLSA tree topology. The loess, spline, and lowess normalizations were four steps distant irrespective of inclusion of the reference taxon. The linear normalization was worse (six steps distant) and the worst result was obtained when the reference taxon was included (eight steps distant).

For the NEU set, no method of analysis recovered the MLSA tree topology, although the loess, spline, and loess normalizations again performed best (two steps distant when binning data at the first quartile). The CGH trees constructed from the linear normalization were at best four steps longer than the MLSA tree when the reference taxon was excluded, and six steps longer when it was included. Binning at the second or third quartile resulted in trees that were typically four steps longer.

For the CON taxon set the MLSA tree topology was recovered only when the reference taxon included, and then only when binning at the first quartile for the spline, loess or lowess normalizations, or at the third quartile for spline and loess normalizations. Other approaches gave trees two to four steps longer than the MLSA tree.

Unlike the BAGEL NJ analyses, which were insensitive to inclusion or exclusion of the reference taxon, the BAGEL parsimony analysis improved when the reference taxon was included in the CGH phylogeny. However, in the BAGEL parsimony analyses, the MLSA tree was recovered only for the CON taxa dataset, any only for a narrow set of approaches, as noted above.

### Treebuilding with MPP, the Microarray to Phylogeny Pipeline (Figure 4, Additional File 4: table S4)

**Figure 4:**
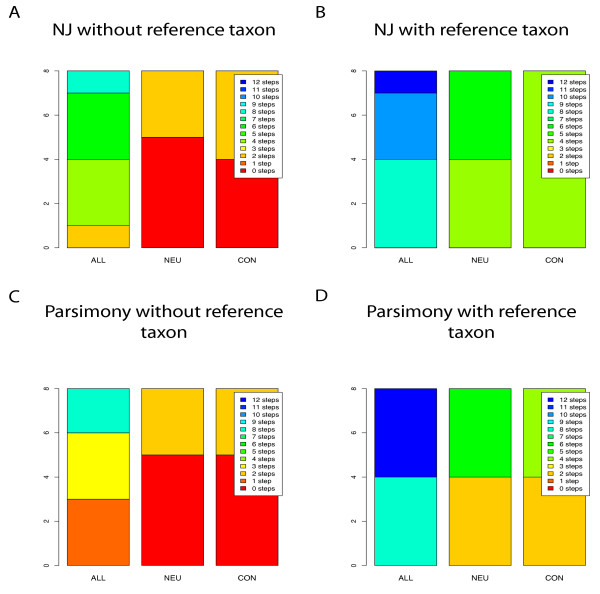
**Results for MPP based NJ and Parsimony Analysis**. The stacked histograms show the SymD measures of trees constructed using the MPP method for the sixteen different iterations detailed in Additional File 1: table S1 - representing the various options available in the MPP program. The binary matrices constructed by MPP were output to PAUP and used to make parsimony-based trees (Figures 4 C and D). From each binary matrix a distance matrix was calculated from each of these sixteen matrices using the cghdist option in the MPP GUI, and a Neighbor-Joining (NJ) tree was calculated using PAUP excluding (Figure 4A) and including (Figure 4B) the reference taxon, *N. crassa *A. This data is also given table form in Additional File 4: table S4.

As described in the methods, we used the MPP pipeline to construct both Neighbor-Joining and Parsimony trees for the three groups of taxa. The MPP method begins by using CGH data to score hybridization probes as present or absent. These data can be exported for parsimony analysis or they can be used to make pairwise distance matrices by a likelihood approach that is designed to compensate for the single reference design. These distance data are then used for phylogenetic analysis by Neighbor-Joining analysis. MPP allows the user to control various options: the CGH data can be transformed using either a log or an inverse hyperbolic sine function (arsinh), the presence or absence of a probe can be estimated by EPP or by BPP, and the binwidth for assigning probe presence or absence can be set at either 0.05 (norm) or determined experimentally (exp). Applying these options in all combinations gave us eight basic combinations of options for both parsimony and NJ phylogenetic analyses.

For the ALL dataset, MPP using NJ did not recover the MLSA tree for any of the eight options. A tree two steps longer than the MLSA tree was recovered using arsinh, BPP, with a binwidth set at norm and excluding the reference taxon. Use of EPP or inclusion of the reference taxon gave trees at least twice as distant.

For the NEU taxon set using MPP and NJ, with the reference taxon excluded, trees concordant with the MLSA tree were recovered for five of the eight options. These five included all of the log-transformed data and the arsinh-transformed data option with BPP and norm binwidth. When the reference was included, the same iterations gave CGH trees four to six steps longer than the MLSA tree.

For the CON dataset using MPP with NJ, the results were similar to the NEU set in that all log-transformed data options recovered the MLSA tree topology when the reference taxon was excluded and no options recovered the MLSA tree topology when the reference taxon was included.

### Parsimony MPP tree construction (Figure 4C and 4D, Additional File 4: table S4)

For parsimony analysis, the same eight combinations were used to convert GCH data to presence/absence data sets and trees were made using 50% Majority-Rule consensus.

For MPP with parsimony analysis of the ALL dataset with the reference taxon excluded, the best trees using any option were one step longer than the MLSA tree (data log-transformed with either EPP or BPP followed by exp binning, or data arsinh-transformed with BPP followed by norm binning). When the reference taxon was included, trees from the four BPP and EPP arsinh-transformed sets were eight steps longer and those from the log-transformed data were even more distant.

For MPP with parsimony analysis of the NEU dataset with the reference taxon excluded, the five iterations that recovered the MLSA tree topology for the NJ analysis did the same for parsimony analysis, i.e., all of the log-transformed data and the arsinh-transformed data option with BPP and norm binwidth. When the reference taxon was included in the CGH phylogeny, no option returned the MLSA topology.

For MPP with parsimony analysis of the CON dataset, when the reference was excluded from the CGH phylogeny, the results were identical to those the results of the NEU taxon set. When the reference was included, the results were nearly identical to those for the NEU data set, i.e., no option returned the MLSA tree topology.

## Discussion

In order to assess if CGH data can be used to infer phylogeny, we applied a variety of approaches to CGH data from nine species of filamentous fungi in the Sordariales derived from a microarray constructed for one of the species, the reference taxon *N. crassa A*. We chose to focus on these taxa because their relationships had been carefully characterized by multi-locus sequence analysis (MLSA) [26-28,42]. The nine species were analyzed in three sets, ALL, NEU and CON, which represent a decreasing range of evolutionary distances. The ALL taxon set has a maximum of 10.5% sequence variation in coding regions [43] and protein amino acid sequence similarity of 60% - 70% [44]. At the other end of the spectrum, the CON taxon set has 2% to 7% sequence difference in coding regions (J. Dettman, unpublished data). We should note that our analysis assumes that hybridization data accurately reflects evolutionary distance. Though the mismatch kinetics of DNA polymorphisms are not always perfectly correlated to hybridization level, the relationship between the two is roughly linear [5,36,45-48]. Consequently hybridization level should provide an adequate proxy for evolutionary distance though it is subject to some experimental noise.

The diversity of approaches that we used to apply CGH data to phylogenetics included the following. To filter the data and normalize them to estimate hybridization levels we used four methods, two from the Acuity package (linear ratio of means, print-tip lowess) and two from the Limma package in R (loess and robust spline). We filtered for pixel saturation and for consistency among replicates. To complement these four approaches, we also used a Bayesian approach (BAGEL) to estimate hybridization levels. Euclidean and correlation methods were used to determine genetic distances from the hybridization levels. The distance method, Neighbor-Joining, was used for phylogenetic analyses of the genetic distances. To allow the use of parsimony phylogenetic methods, genetic distances were converted to binary data using GACK. We also investigated the microarray-to-phylogenetics pipeline (MPP), which transforms the data with either of two methods (log or arsinh) and converts the hybridization levels to binary data by either of two methods (EPP or BPP) for use in parsimony analysis. The binary data are then converted to genetic distances using a likelihood method intended to compensate for the shortcomings of using a single reference taxon for CGH. To assess the utility of the many permutations of these methods, we compared phylogenetic trees made from CGH data to the MLSA tree for the sordariaceous fungi using both symmetric distance (SymD) and taxon pruning (D1).

We found no single method that consistently produced a CGH phylogeny equivalent to an MLSA phylogeny. Instead, all the methods had different degrees of success depending on the combination of treatments applied to the data. Two trends stood out: that the greater the genetic distance among taxa the lower success, and that distance phylogenetic analysis, Neighbor-Joining, performed better than parsimony analysis. However, even with distance methods and data sets with restricted genetic distance, success was low; the NJ trees the NEU and CON topologies were recovered 20.6% and 25% of the time, respectively. It should be noted that the greatest distance among taxa was only 10.5%, roughly at the acknowledged limit of utility for long oligomer arrays [45].

There was considerable variation between the normalization methods. For the distance-based trees, the most successful recovery was with a basic linear normalization (25% overall) and the worst was lowess normalization (6.25%). For the parsimony trees, the linear normalization was the worst, with a 6.3% recovery rate and the best was the robust spline, with a recovery rate of 12%.

The Neighbor-Joining method was better than the parsimony method (15% vs. 6.93% recovery). Of the two distance metrics used to construct distance trees, the Euclidean method performed better than the correlation-based metric (21% v. 9.8%). This result was in contrast to our *in silico *investigations, detailed in the accompanying work, where the correlation-based metric was superior in capturing topologies. The Euclidean metric, however, was better able to estimate the branch-lengths. Typically, correlation measures are more reliable as they smooth rough data. However, in this case correlation measures may not be sensitive enough to distinguish very small differences in relationships based on microarray data, particularly with taxa close to the reference taxon.

When hybridization levels for the CON and NEU datasets were estimated using BAGEL, distance phylogenetic analysis recovered the MLSA tree more often, but this advantage was not seen with parsimony analysis.

Tree construction with Bayesian estimates of relative hybridization levels for each species was slightly more robust than a simple average or median of ratio values. For the distance trees of the CON and NEU datasets, more BAGEL-treated normalizations recovered the MLSA tree than normalization alone. The advantage of BAGEL-treated datasets was not seen with parsimony analysis.

### MPP NJ and Parsimony

The MPP platform, an all-inclusive pipeline designed specifically for CGH phylogeny construction, performed similarly to the traditional filtering and normalization methods. MPP was sensitive to inclusion of the reference taxon, that is, no NJ tree equalled the MLSA tree when the reference taxon was included, but 37.5% of NJ trees and 41.7% of parsimony trees did equal the MLSA tree with the reference taxon was excluded. MPP was also sensitive to the genetic distance among taxa, no analysis of the ALL data set found the MLSA tree, while the MLSA tree was recovered from the CON and NEU datasets in between 25% and 32% of NJ or parsimony analyses. In MPP, the Log transformation, with a fixed binwidth, performed better than the arsinh transformation, indicating that the former is better suited to our empirical CGH data.

Our analysis of CGH for eukaryotic microbes may be compared to a similar study of yeast species [1]. We took the opportunity to reanalyze the yeast CGH data with the newly developed MPP workflow [2] to see if our *Neurospora *results were of general significance. The yeast dataset contains more genetic distance than the *Neurospora *dataset (the two closest taxa, *S. cerevisiae *and *S. paradoxus*, differ by 15% [49-51]), so the analysis of yeast data would be expected to be more challenging. CGH phylogenies were compared to a MLSA phylogeny for the yeast taxa based on 106 orthologous genes [50]. When all eight taxa (including the reference species, *S. cerevisiae*) from the CGH study of Edwards-Inghram et al. [17] were used for tree construction, all CGH trees were at least ten steps longer than the MLSA tree. The gap dropped to 8 steps when *S. cerevisiae *was excluded (data not shown). When the taxon set was restricted to just the six species studied by both Edwards-Inghram and Rokas et al. (i.e., excluding the two taxa closest to the reference taxon, *S. cariocanus *and S. *boulardii*), some CGH trees equalled the MLSA tree. Here, BPP outperformed EPP in converting CGH data to discrete data, although both methods did equally well when the reference species was excluded. It seems that MPP analysis of yeast is confounded by taxa that are too closely related, whereas MPP analysis of *Neurospora *was confounded by taxa that were too distantly related. In the end, however, with neither yeast nor *Neurospora *could CGH data be counted on to recover the sequence-based MLSA phylogeny reliably or consistently.

As a final point of discussion, our results with empirical CGH data can be compared to our previous analyses of simulated CGH data, which allowed for comparison of three different topologies. In both cases, distance analysis was superior to parsimony analysis, probably due to the loss of information when genetic distances are converted to discrete data. Similarly, using distance analysis, highly filtered data sets produced less well-resolved phylogenies than data sets that included more ratio data. Finally, it was more difficult to recover MLSA phylogenies using empirical CGH data than using simulated CGH data, likely due to the additional noise in empirical data.

## Conclusions

Our results with empirical CGH data and those of the accompanying *in silico *analysis demonstrate that aCGH-based phylogenetics cannot be counted on to produce a phylogeny equivalent to those derived by MLSA. Even with methods specifically designed to compensate for the single reference CGH design, there is inconsistent recovery of the MLSA phylogeny. Therefore, in experimental contexts without prior knowledge of the relationships, it would be impossible to be certain that the true tree was recovered by CGH phylogeny. These findings are in contrast to several published works in bacterial species, which have found concordance between their aCGH tree and those based on one or a few loci. While our results confirm that aCGH is partially successful in some cases, inconsistent recovery in our datasets precludes our endorsement of the technique for widespread use. This does not forestall the usefulness of CGH data for various other analyses.

Our analysis does suggest that some normalization and post-processing methods may best reflect the underlying genetic distance between taxa and these methods might be best for other analyses of CGH data. Of the normalization methods implemented, the linear and robust spline methods worked better than the lowess/loess methods. The BAGEL estimation of hybridization levels also performed well. Unlike most other methods, it allowed for inclusion of the reference without a penalty. If a quick approximation of a topology is sufficient for the user's needs, the MPP pipeline offers a simple and easy way to construct a tree from CGH data. However, even the MPP approach recovered the MLSA topology less than half the time. If phylogeny is the aim, it would be better to invest in a modest MLSA approach.

## Authors' contributions

LBG participated in its design and coordination and drafted the manuscript. LG, TK, and JWT also participated in the design of the study. LBG modified existing code to automate programs used in this work. LBG completed the statistical analysis. LBG and JWT wrote the manuscript. All authors read and approved the final manuscript.

## Supplementary Material

Additional file 1 **Additional table S1 - **Method matrix listing data treatments that are represented in each figure. Click here for file

Additional file 2 **Additional table S2 - Figure 2: Neighbor-Joining and GACK Parsimony Analysis when the dataset is only normalized**. Figure 2 in table form, with actual tree scores. Zeros are bolded. In excel format. Click here for file

Additional file 3 **Additional table S3 - Figure 3: NJ and Parsimony Analysis after Bayesian estimation of a relative hybridization level**. Figure 3 in table form, with actual tree scores. Zeros are bolded. In excel format. Click here for file

Additional file 4 **Additional table S4 - Figure 4: MPP-Based Tree Construction**. Figure 4 in table form, with actual tree scores. Zeros are bolded. In excel format. Click here for file
